# Cognitive evoked potentials and central auditory processing in children with reading and writing disorders

**DOI:** 10.1590/S1808-86942012000300016

**Published:** 2015-10-14

**Authors:** Gislaine Richter Minhoto Wiemes, Lorena Kozlowski, Marcos Mocellin, Rogério Hamerschmidt, Luiz Henrique Schuch

**Affiliations:** a MSc in Communication Disorders at Universidade Tuiuti do Paraná (UTP/PR) (Speech and Hearing Therapist at Hospital de Clínicas of the Federal University of Paraná - HC/UFPR).; b Post-doctoral degree at the University of Montreal - Canada. (Speech and Hearing Therapist, Professor in the MSc and PhD programs on Communication Disorders at UTP/PR).; c PhD, Professor in the Department of Ophthalmology and Otorhinolaryngology at HC/UFPR. (Professor in the Department of Ophthalmology and Otorhinolaryngology at HC/UFPR).; d MSc, Assisting Professor in the Department of Ophthalmology and Otorhinolaryngology at HC/UFPR. (Assisting Professor in the Department of Ophthalmology and Otorhinolaryngology at HC/UFPR).; e MD (Resident Physician on Otorhinolaryngology at Hospital de Clínicas of the Federal University of Paraná). Hospital de Clínicas da Universidade Federal do Paraná.

**Keywords:** attention deficit disorder with hyperactivity, event-related potentials, hearing, p300

## Abstract

Learning disorders are often magnified by auditory processing disorders (APD).

**Objective:**

This paper aims to verify whether individuals with reading and writing disorders and P300 latencies above the average also present altered Staggered Spondaic Word (SSW) and speech-in-noise test results suggestive of APD.

**Materials and Methods:**

This is a cross-sectional cohort study. Twenty-one individuals with reading and writing disorders aged between 7 and 14 years were enrolled.

**Results:**

All subjects had normal findings on ENT examination, audiological tests, and brainstem auditory evoked potentials. The average P300 latency (334,25 ms) of all patients was picked as a cutoff point to divide the subjects into two groups: group A with latencies above 335 ms, and group B with latencies below 335 ms. Individuals in group A underwent SSW and speech-in-noise testing.

**Conclusion:**

Altered results in the SSW and speech-in-noise tests suggestive of APD were found in the group of individuals with reading and writing disorders with P300 latencies above 335 ms.

## INTRODUCTION

Children with learning disorders are often referred to otorhinolaryngologists by schools, speech and hearing therapists, pedagogues, psychologists, pediatricians, and pediatric neurologists to have their hearing assessed and find whether some form of hearing loss is compromising their performance at school.

The assessment usually comprises an interview, otoscopic examination, and peripheral auditory pathway testing (pure-tone audiometry, speech discrimination and acoustic impedance testing) and results are mostly within normal ranges. Thus, learning disorders are not associated with hearing loss.

Good hearing depends on the integrity of the auditory pathways together with the entire auditory system, from the outer ear to the cortex.

Quality of hearing can only be assessed when auditory processing disorder (APD) manifestations are analyzed through a set of specific tests that look into central auditory function.

The investigation includes data on family history, otoscopic examination, peripheral auditory pathway testing, and seldom-performed central auditory pathway tests such as auditory potential and cognitive evoked potential (P300) tests.

Speech recognition disorders magnify language development disorders and affect the development of school skills^1^. Learning and auditory processing disorders are frequently found in classrooms^2^.

The first studies on central auditory disorders to draw attention to the use of devices to modify or distort verbal messages to diagnose central deafness were published by Booca et al.^3^. After being detected in the inner ear, sound goes through a series of physiological and cognitive processes to be decoded and comprehended^4^.

Kimura^5^ uses staggered spondaic word tests to assess central auditory system function in patients with known injuries to relate the difficulties reported by the patients to the site of injury in the central nervous system.

Central auditory function integrity is often assessed through the Staggered Spondaic Word (SSW) test designed by Katz^6^ to elicit complex responses and provide qualitative and quantitative bases for hearing analysis. This test uses binaural material and two types of auditory tasks, non-competitive or diotic and competitive or dichotic. Non-competitive tasks present equal simultaneous tones to both ears, while competitive tasks present two different tones at the same time, one to each ear^7^. Machado^1^ adapted the test for the Portuguese language, but due to the relative scarcity of spondaic words in our language they were replaced by grammatical locutions, short phrases, and slang to validate the Portuguese version of the SSW test.

Central auditory hearing loss in children is also verified through the speech-in-noise test. This test compares the subject's speech-in-noise recognition levels of both ears by presenting one-syllable and two-syllable words at various degrees of noise intensity.

The P300 cognitive evoked potential electrophysiological test presents a long latency positive potential generated in the auditory association areas about 300 ms after the presentation of a tone. This test reflects the subject's cognitive skill level and verifies whether disorders are present in the auditory association cortex.

This electrophysiological test can be performed to objectively assess the integrity of the cortical auditory pathways. The P300 component can be obtained for a series of stimulation conditions in which individuals are asked to process relevant task-related information. When individuals are able to consciously recognize the occurrence of a change in the tones - an odd tone played after a series of other tones following a pattern - a broad positive component is generated with a latency of approximately 300 ms^8^, ^9^.

The use of the P300 test in adults and children lacks standardization to account for the different levels of development of their central auditory systems, and is still not part of the medical research protocol adopted to study learning disorders and attention deficit. However, it has been considered useful in the studies on memory disorders, sequential information processing, and decision making^9^.

Peripheral auditory function is usually normal in cases of auditory processing disorder. Consequently, traditional peripheral auditory function assessment brings little or no information on central auditory perception.

Children with learning, reading, language acquisition, attention, and hyperactivity disorders may present altered results in auditory processing (AP) tests^10^.

This paper aims to identify whether a group of individuals with writing and reading disorders presenting above-the-average P300 latencies also have staggered spondaic word (SSW) and speech-in-noise test results suggestive of auditory processing disorder.

## MATERIALS AND METHODS

This is a contemporary cross-sectional cohort study.

This study was approved by the Human Medical Research Ethics Committee and given permit number 249.053/2000-08, BANPESQ nº 20006692; it complies with the standards set in Resolution 196/96 from the Ministry of Health.

Twenty-one individuals aged between 7 and 14 years were enrolled in the study. Twelve (57.1%) were males and nine (42.9%) were females.

The subjects were assessed by a multidisciplinary team (pediatric neurologist, pedagogue, psychologist, linguist, and speech and hearing therapist) under the diagnostic assumption of specific reading and writing disorder (letter inversions, problems with left-to-right orientation, dysgraphia, dyslexia, dysorthography), along with attention deficit and hyperactivity disorder, as compounded factors leading to learning disorder. Individuals who failed to pass school years were enrolled.

Enrollment criteria included: referral to the ENT ward, normal ENT assessment, normal pure-tone thresholds, acoustic impedance measurements showing A-shaped tympanograms, static compliance within normal range, present acoustic reflexes, and preserved auditory pathways as verified by brainstem evoked auditory potential testing.

Cognitive evoked potential tests were done using a NIHON KODEN Neuropack 2 device with electrodes connected to the following sites: forehead – ground (Fpz), vertex (Cz) – both in the middle line and ipsilateral (A1) and contralateral (A2) earlobes. The subjects were advised to hear two tones in different frequencies (750 Hz and 2000 Hz), one played more often than the other in a 4:1 ratio at 70 dBNA, and listen for the rarer tone (2000 Hz). Tones were presented in a binaural fashion and responses to frequent and rare tones were measured separately. P300 is an endogenous potential that occurs approximately 300 ms after the tone is played. It may be bimodal and present two peaks (“a” and “b”), “a” being apparently of frontal origin and occurring some 275 ms after the tone is played, and “b” with parietal representation occurring some 300 ms after the tone is played^9^. Subjects with both peaks had peak “b” considered in their assessment.

Groups were divided based on the P300 results. A cutoff point of 335 ms was picked, as the average P300 for the right and left ears of the 21 individuals enrolled in the study was 334.25 milliseconds. Ten individuals with P300 latency above 335 ms were included in group A, while 11 individuals with P300 latencies under 335 ms on both sides were placed in group B.

Table 1 presents a description of groups A and B containing individuals with reading and writing disorders based on P300 cognitive evoked tests.

**Table 1:** Average P300 for groups A and B – RE and LE.

A	A	B	B
P300 – RE	P300 – LE	P300 – RE	P300 – LE
314	338	304	309
342	329	303	318
380	385	267	270
427	416	287	330
428	396	279	279
348	345	331	331
384	384	317	314
387	407	301	301
340	328	309	297
314	339	318	307
		279	292

366.4	366.7	299.5	304.4

RE – right ear; LE – left ear.

The data gathered for groups A and B were statistically treated through the Wilcoxon test to allow the analysis of the differences in latency between right and left ears in each group; the Mann-Whitney test was used to analyze the latency differences between groups A and B for both ears, and the average P300 latency found for each ear in each group and between groups.

Individuals with P300 latencies greater than 335 ms were piked to undergo speech-in-noise and SSW testing.

Speech-in-noise and SSW tests were carried out in a soundproof booth using a two-channel MAICO KS5 audiometer; a CD containing Auditory Processing^11^ tests was played.

Speech-in-noise tests were done at signal/noise ratios of 0 dB, -10 dB and – 20 dB in contralateral presentation, and test intensity was based on the audiogram averages at 500 Hz, 1 kHz and 2 kHz, +40 dB, with white noise at the same level. Percent values were captured, and results were considered normal for subjects getting at least 80% of right answers on each list.

SSW tests were performed at 50 dBNS, above the average of 500 Hz, 1 kHz and 2 kHz. The method developed by Katz^6^ was used to standardize test scores.

## RESULTS

The Wilcoxon and Mann-Whitney tests were used in the statistical treatment given to P300 latency data sets. Average P300 latency was also calculated. In-group P300 wave latencies of right and left ears were compared, as were the latencies found in groups A and B.

The Wilcoxon test showed no statistical difference in the right and left ear latencies in members of the same group. The Mann-Whitney test revealed statistical difference between the latencies observed in groups A and B for both ears.

Statistically significant differences were see when the P300 latency average was used as a cutoff to separate groups A and B. Subjects in group A averaged 366.4 ms on right-ear latency and 366.7 ms on left-ear latency, and had a combined average of 366.5 ms. Group B members averaged 299.5 ms on right-ear latency and 304.4 ms on left-ear latency, and had a combined average of 301.9 ms. No significant differences were found when ears within the same group were compared for P300 latency.

This study did not aim at studying P300 wave amplitudes, thus we may state that values ranged between 1.7 and 20uV^12^ as seen in the literature, regardless of the group the subject belonged to. P300 studies done on similar populations do not use P300 amplitude as a parameter in clinically differentiating groups^13^, and consider latency as a more important parameter to analyze results^14^, ^15^.

Test results were analyzed based on the percent occurrence of speech-in-noise and SSW test outcomes.

In speech-in-noise tests the ipsilateral and contralateral messages were elicited at signal/noise ratios of -10/0 dB and -20/0 dB for spoken messages. One (10%) of the selected individuals did not do the test. Seven (70%) subjects had hearing disorders according to the test, specifically in what concerns selective attention and auditory closure. Two (20%) individuals had normal latencies inconsistent with hearing disorder.

Three (42%) of the seven subjects with speech-in-noise findings consistent with auditory disorder had higher levels of involvement in the right ear, while two (29%) had worse test results in their left ears, and two (29%) had both ears equally involved.

The interpretation of SSW test results indicated that seven (70%) individuals had severe APD, two (20%) had moderate APD, and one (10%) had mild APD.

The classification for APD indicates that the compromised auditory skills revealed in the tests pointed to altered decoding, encoding, and organization capabilities.

Ten of the 21 individuals tested in our study had P300 latencies above 335 ms and were submitted to speech-in-noise and SSW testing. Seven (70%) of these 10 individuals had test results consistent with hearing disorder in the speech-in-noise test, and the same ten (100%) had test results consistent with AP disorder in the SSW test.

Test result analysis indicated that the subject with the worst P300 latency average for both ears (421.5 ms) also had the worst score in the speech-in-noise test. This subject had test results consistent with auditory disorder, equal involvement of both ears, and SSW findings suggestive of severe APD.

The opposite was found in relation to the subject with the best P300 latency average (326.5 ms): both ears performed equally in the speech-in-noise test and no central auditory disorder was identified, while SSW findings were suggestive of mild APD.

P300 test results were suggestive of APD starting from 335 ms. However, as individuals with below-the-average P300 were not tested, it is not clear whether group B test results would be any better. All individuals with latency above 335 ms had altered SSW test results and 70% of them had speech-in-noise test results consistent with APD. No statistical differences were found in relation to the ear to which 2000-kHz tones were presented. Statistically significant difference was seen in P300 wave latencies between groups A (latencies above 335 ms) and B (below 335 ms).

## DISCUSSION

The individuals enrolled in this study diagnosed with specific reading and writing disorder had the same social-economic and cultural status, as these variables could affect some AP skills, as in order to be successful at school one needs to be able to properly process auditory information^15^, ^16^.

Pure-tone audiometry and acoustic impedance tests were carried out to rule out the presence of outer, middle, and inner ear disorders that could affect the results of cognitive evoked potential, SSW, and speech-in-noise tests.

The purpose of this paper was to identify whether a group of individuals with reading and writing disorders and above-the-average (334.25 ms) P300 latencies also had altered SSW and speech-in-noise test results consistent with auditory processing disorder. Therefore, these tests were not offered to all enrolled individuals. The Mann-Whitney test revealed latency differences between groups A (average 366.5 ms) and B (average 301.9 ms).

Auditory processing refers to how effectively and efficiently the central auditory nervous system utilizes auditory information^17^, i.e., AP conjoins a series of specific skills individuals depend on to understand what they hear. This study indicates that individuals with normal pure-tone audiometry test results claiming they are able to hear but not to understand what they hear should undergo specific AP testing, once auditory processing is a cerebral function studied as a multidimensional response to stimuli presented through the auditory pathways^4^.

Studies done on P300 results of patients with injury in auditory areas of the brain have indicated that P300 latencies and amplitudes were significantly different in patients with injured auditory areas when compared to normal control group subjects^18^.

P300 is a cognitive endogenous potential, as it reflects the functional use subjects make of auditory stimuli, regardless of the subject's physical characteristics. This study covered 21 patients aged between 7 and 14 years with an P3 wave average latency of 334.25 ms. A study done with 20 healthy young adults showed a P3 wave average latency of 310.92 ms. Latency and amplitude values varied considerably in the studied population^19^.

All subjects included in our study complained of poor school performance. Individuals in group A had greater P300 latencies and presented altered SSW and speech-in-noise test results, as also seen in studies on P300 results in children without signs of APD and children diagnosed with APD, in which longer latencies were observed in the group of children with APD^20^, ^21^, ^22^, ^23^, ^24^, ^25^.

Significant variability was seen in P300 results, and individuals with lower latency also performed better in AP tests^25^.

We agree with the explanation provided by Aquino et al.^24^ in that latencies above normal could be due to neuronal immaturity, given up to the age of 15 latencies tend to diminish. Conversely, P300 latencies above normal levels are indicative of out-of-the-norm cognitive processing. Studies have shown that ADHD patients have greater latencies and lower P300 amplitudes when compared to control subjects, possibly due to the presence of less mature central auditory pathways^26^.

P300 allows the assessment of how long it takes for sound to be perceived and interpreted by the auditory cortex and to identify individuals with cognitive disorders. Despite its low specificity, P300 may be used both to assess and follow up subjects with cognitive disorders.

This study and others done on children with learning disorders used speech-in-noise and SSW tests to reveal the presence of auditory disorders and compromised hearing skills^27^, the latter also reflected on P300^28^, ^29^.

Electrophysiological tests (P300) were proven more sensitive but are non-specific, while SSW and speech-in-noise tests for APD were more specific and relevant in defining the proper speech and hearing therapy to be offered to subjects with altered auditory skills^24^, ^30^, ^31^.

APD has been frequently seen as the cause for learning disorders. A multidisciplinary approach is required to assess the integrity of the perceptual, linguistic, and cognitive systems^10^, ^27^, ^32^. The differential diagnosis must include disorders such as attention deficit hyperactivity disorder (ADHD), language disorders, reading and writing disorders, learning disorders, psychical disorders, and mild mental disease, as they may be confused with AP alterations and these disorders may coexist^33^.

AP behavioral assessment assists in the diagnosis as it reveals the impaired auditory skills and is useful in following up the development of individuals enrolled in speech and hearing rehabilitation programs^34^. Additionally, it allows for the assessment of outcomes before and after rehab. This point is better illustrated with a case^35^, in which clear goals concerning the impaired skills were devised after objective and behavioral testing. AP test result analysis enabled the development of a comprehensive strategy to cover individual specific goals. The process was managed by a trained expert, included orientation to the subject's family and teachers, and offered adequate therapy to address the individual's auditory impairments. Proper auditory training led to improved medical test results, more satisfactory performance levels at school, and better engagement with the family, showing that improvement after auditory training is related to changes in the central auditory nervous system.

P300 cognitive evoked potentials offer significant screening capabilities in the selection of individuals requiring behavioral testing and assessment of specific auditory skills for the purposes of developing targeted rehabilitation programs. This system may also be used for individuals with multiple defects for whom AP improvements might positively affect their attention, focus, learning experience, and level of activity.

## CONCLUSION

This study revealed that individuals with writing and reading disorders with P300 latencies above 335 ms had altered results in staggered spondaic word (SSW) and speech-in-noise tests suggestive of auditory processing disorders.
